# Albumin and Furosemide Combination for Management of Edema in Nephrotic Syndrome: A Review of Clinical Studies

**DOI:** 10.3390/cells4040622

**Published:** 2015-10-07

**Authors:** Margaret Duffy, Shashank Jain, Nicholas Harrell, Neil Kothari, Alluru S. Reddi

**Affiliations:** Department of Medicine, Rutgers New Jersey Medical School, 185 South Orange Avenue, Newark, NJ 07103, USA; E-Mails: md13086@gmail.com (M.D.); jain.shashank.3@gmail.com (S.J.); nbharrell@gmail.com (N.H.); kotharne@njms.rutgers.edu (N.K.)

**Keywords:** nephrotic syndrome, albumin and diuretic, diuresis, natriuresis, edema management

## Abstract

The treatment of edema in patients with nephrotic syndrome is generally managed by dietary sodium restriction and loop diuretics. However, edema does not improve in some patients despite adequate sodium restriction and maximal dose of diuretics. In such patients, combination of albumin and a loop diuretic may improve edema by diuresis and natriuresis. The response to this combination of albumin and a diuretic has not been observed in all studies. The purpose of this review is to discuss the physiology of diuresis and natriuresis of this combination therapy, and provide a brief summary of various studies that have used albumin and a loop diuretic to improve diuretic-resistant edema. Also, the review suggests various reasons for not observing similar results by various investigators.

## 1. Introduction

Edema is a common clinical symptom in patients with nephrotic syndrome. The nephrotic syndrome in adults is characterized by proteinuria (>3.5 g/1.73 m^2^), hypoalbuminemia, edema, hyperlipidemia, and lipiduria. In children, nephrotic syndrome is defined as proteinuria >40 mg/m^2^/h or 1st voided urine protein:creatinine ratio >2–3 (mg/mg). However, the patient notices edema early on, and this symptom primarily brings him/her to the attention of a clinician. The initial management of edema is sodium restriction (approximately 2 g sodium or 88 mEq a day in adults) and diuretic therapy. The most common diuretics used clinically are loop diuretics (furosemide, bumetanide, or torsemide) with addition of metolazone, or spironolactone, as needed. When both sodium restriction and diuretic therapy fail, albumin is added to improve intravascular volume, diuresis and natriuresis. The combination of a loop diuretic (furosemide) and albumin has resulted in decrerased edema, particularly in patients with hypoalbuminemia. The practice of this combination therapeutic approach has been extended to patients with congestive heart failure and liver cirrhosis. The rationale for this combination is two-fold. First, this combination is assumed to improve diuresis, natriuresis, and reduction in body weight; and second is the improvement in the delivery of loop diuretics to their site of secretion. The beneficial effects of the combination of albumin and furosemide are briefly discussed below.

## 2. Diuresis and Natriuresis

It is well established that both diuretics and albumin will induce diuresis and natriuresis in patients with edema and hypoalbuminemia. It is also known that the diuretic response is suboptimal in some patients with hypoalbuminemia despite the use of high doses alone or in combination with other diuretics. This blunted response seems to be related to relatively low intravascular volume and associated activation of neurohumoral (sympathetic nervous system and antidiuretic hormone) and renin-angiotensin II-aldosterone systems. Some studies have shown that albumin infusion alone induced diuresis and natriuresis [1] possibly by increasing intravascular volume with suppression of the above hormonal systems. However, the response to albumin has not been uniformly observed. High doses of diuretics alone or in combination with other diuretics may have several metabolic effects besides ototoxicity. For this reason, it is the belief of many clinicians to combine low to moderate doses of loop diuretics with albumin to induce diuresis, natriuresis, and loss of body weight. Indeed, Davison *et al.* [1] reported a weight loss of 27 kg in 14 days in a severely edematous patient with nephrotic syndrome who had recently undergone appendectomy with resultant abdominal wall edema as well.

In nephrotic patients, good response to the combination of furosemide and albumin is assumed to be related to repletion of intravascular volume. A review of plasma volume status in untreated nephrotic syndrome patients stated that one-third of patients had low plasma volume, normal in one-half, and increased in the remainder of patients [2]. A similar study found either normal or increased plasma volume in nephrotics with the exception of patients with minimal change disease [3]. In some of these patients, the plasma volume may be low with high adrenergic and renin activity. The routine use of albumin alone in nephrotic patients with normal to increased plasma volume is questioned by many investigators. In order to achieve sufficient diuresis and natriuresis in nephrotic syndrome patients, some investigators used the combination of furosemide and albumin, and reported variable results.

## 3. Diuretic Delivery to the Proximal Tubule

Loop diuretics such as furosemide are highly albumin bound (>90%), and therefore, furosemide is not filtered at the glomerulus. However, it is secreted in the proximal tubule via an organic acid transporter into the lumen. In a patient with hypoalbuminemia (serum albumin concentration <2 g/dL), furosemide is less bound [4] to albumin and the free drug diffuses into the tissues with resultant increase in its volume of distribution. This results in less delivery to the proximal tubule for secretion into the lumen. In order to deliver a substantial amount of the drug to the proximal tubule, mixing furosemide with albumin is assumed to improve the delivery for its secretion. In clinical practice, two approaches have been adapted to improve the delivery. One is premixing albumin with furosemide, and the other is to administer 25–50 g of albumin followed by infusion of furosemide 40–80 mg. Even if furosemide is secreted in the proximal tubule, some of the drug is bound to the filtered albumin in the tubular lumen. This binding with albumin in the lumen makes for less free drug delivery to the thick ascending limb of Henle’s loop for its action on the Na/K/2Cl transporter. Thus, diuretic resistance develops from not only low dose delivery but also from its binding to the filtered albumin in the tubular lumen. Other causes such as hypertrophy of distal tubular cells due to increased reabsorption of delivered NaCl have been implicated in diuretic resistance. The pharmacokinetic and pharmacodynamic studies are mixed with the combination of furosemide and albumin. In this narrative review, we will summarize the available studies on combination therapy in patients with nephrotic syndrome.

## 4. Clinical Studies Using the Combination Therapy for Edema Management

As mentioned above, albumin has been used to improve edema in patients with nephrotic syndrome in addition to diuretics [1]. However, the improvement in edema was not observed in all patients. Also, as mentioned above, the failure to observe improvement in edema with diuretics alone has been attributed to diuretic resistance. These observations led Inoue and colleagues [5] to evaluate the efficacy of combination therapy in analbuminemic rats and hypoalbuminemic patients. These investigators demonstrated a significant increase in urine output in analbuminemic rats when furosemide and an equimolar amount of albumin were administered together, as compared with either furosemide or albumin alone. In contrast, the fractional excretion of furosemide was significantly lower in analbuminemic rats compared to normal rats when furosemide was given alone, suggesting diffusion of furosemide into various tissues. This distribution into tissues was decreased when furosemide was combined with albumin, with resultant decrease in furosemide excretion.

Inoue *et al.* [5] also reported similar results in human subjects with hypoalbuminemia due to a variety of causes, including cirrhosis and chronic renal failure. The combination of albumin and furosemide produced an increase in urine output ranging from 31–245 mL/h when compared to furosemide or albumin alone. Maximal effect was noted at one hour after administration with diminished effect thereafter. This study suggested that administration of albumin to analbuminemic or hypoalbuminemic patients causes furosemide-albumin binding and delivery of this complex to the proximal tubule for secretion of furosemide into the tubular lumen. A case report by Mattana *et al.* [6] suggests that the combination of albumin and furosemide induced diuresis and natriuresis in a patient with nephrotic syndrome and chronic renal failure, but the patient died because of upper gastrointestinal hemorrhage. Owing to promising results with combination therapy, more clinical trials were pursued in subsequent years.

Baddington *et al.* [7] reported in five diuretic resistant patients with nephrotic syndrome that the combination of albumin and furosemide caused sustained diuresis, natriuresis, and weight loss for 1 week. The serum creatinine levels, which were deteriorating prior to treatment, improved after combinations therapy. These investigators suggest that combination therapy improves edema in those patients who are diuretic resistant.

In 1995, Akcicek *et al.* [8] reported the first controlled study that compared the effect of furosemide with furosemide and albumin in the same patient. Eight patients with nephrotic syndrome and grossly apparent clinical edema were given three treatments with albumin alone, furosemide alone, and a furosemide-albumin combination. Clinical endpoints were urine volume and excretion of sodium and potassium prior to, 18 h, and 24 h after treatment. The study did not find any benefit of the combination of furosemide and albumin.

In 1999, Fliser *et al.* [9] compared the effects of the combination of furosemide and albumin, furosemide, or albumin alone in nine nephrotic patients on urine volume, urinary sodium, urinary chloride, and serum albumin, atrial natriuretic factor (ANP), mean arterial pressure (MAP), glomerular filtration rate (GFR), and effective renal plasma flow (ERPF). The results showed an increase in urine volume, sodium excretion, and chloride excretion at 8 and 24 h after infusion of both albumin and furosemide when compared to either furosemide or albumin alone. Also, increases in both ANP and ERPF were observed, suggesting that combination therapy is superior to either furosemide or albumin administration alone.

In 2001, Na *et al.* [10] studied the effects of albumin and furosemide versus furosemide alone in seven nephrotic patients. Albumin was given 60 min prior to furosemide. Clinical endpoints were urine volume, urine sodium and chloride, and osmolal as well as free water clearance at 24 h. Results were extrapolated to calculate pharamacokinetic data (total plasma clearance, volume of distribution, elimination half life, and urine furosemide excretion). The authors found that albumin pre-infusion modestly increased diuresis but not natriuresis with no effect on the pharmacokinetic properties of furosemide.

Ghafari *et al.* [11] in 2011 published a study on 10 nephrotic patients with reported normal kidney function. Treatment groups included albumin alone, furosemide alone, or furosemide-albumin complex. Clinical outcomes were urinary output and sodium excretion at 24 h. Their work demonstrated that co-administration of albumin and furosemide caused an increase in urine volume and sodium excretion when compared to furosemide alone.

In 2012, Phakdeekitcharown *et al.* [12] studied the effects of furosemide versus combination of albumin and furosemide in 24 patients with hypoalbuminemic chronic kidney disease. A submaximal dosage of furosemide (40 mg) was used. Urine volume, urinary sodium, urinary potassium, blood pressure, calculated GFR, and serum albumin were used as clinical endpoints, which were measured at 6 and 24 h after administration. The results of their study showed a short-term (6 h) beneficial effect in albumin combined with furosemide versus furosemide alone in both diuresis and natriuresis. They further stated that their findings were consistent with the hypothesis that albumin may assist furosemide delivery to its site of action and increase renal blood flow in patients with hypoalbuminemia.

**Table 1:** Clinical studies assessing use of furosemide and albumin in patients with nephrotic syndrome.

Study	Design	No. of Patients	Mean Age (Year)	Disease	Serum Albumin (g/dL)	Serum Cr (mg/dL)	Furosemide (Fu)-Human Albumin (A) Dose	Results *
**Adults**								
Eadington *et al.* [7]	Observational	5	55.4	Nephrotic syndrome (NS)	1.5–2.6	2.86	NR	Significant weight loss at 1 week
Akcicek *et al.* [8]	RCT, cross-over	8	NR	Nephrotic syndrome (NS)	1.1–2.2	1.2–2.4	Fu: 220 mg total (60 mg bolus + 40 mg/h × 4 h) A: 0.5 mg/kg × 4 h	No increased efficacy of combined F-A compared to F alone
Fliser *et al.* [9]	RCT, cross-over	9	48 ± 4	NS with biopsy-proven renal disease	3.0 +/− 2.3	All < 1.3 except in one patient (NR)	Fu: 60 mg A: 200 mL of 20% soln.	Increase in 8 h urinary volume, U_Na_, U_Cl_, and U_alb_ in F-A when compared to either alone
Na *et al.* [10]	RCT, cross-over	7	41 ± 23	NS with biopsy-proven renal disease	1.7 +/− 0.2	1.6 ± 0.8	Fu: 160 mg A: 100 mL of 20% soln.	Combined F-A increased diuresis but not natriuresis
Ghafari *et al.* [11]	RCT, cross-over	10	NR	NS	NR (<3.5)	NR (stated “normal kidney function”)	Fu: 2 mg/kg/TDS A: 0.5 g/kg/TDS	Increase in 24 h urine volume, FeNa, and urine sodium in F-A when compared to either alone
Phakdeekitcharoen *et al.* [12]	RCT, cross-over	24	66.4 ± 12.8	Hypoalbu-minemia and CKD (GFR <60 mL/min)	3.0 +/− 0.3	2.2 ± 0.8	Fu: 40 mg A: 10 g of 20% soln.	Combined F-A had superior short-term (<6 h) diuretic and natriuretic effect compared to F alone
**Children**								
Weiss *et al.* [13]	Cohort	24	NA	NS	1.8 ± 0.3	NA	Fu:1–2 mg/kg A: ~1 g/kg	Reduction in body weight (not compared with furosemide or albumin alone)
Haws and Baum [14]	Retrospective	21	5.5 ± 0.5	Primary glomeular diseases	1.6 ± 0.2	0.7 ± 0.1	Fu:1.5 mg/kg A: 25%	Body weight loss of 1.2% ± 0.2% per infusion
Bircan *et al.* [15]	Prospective	14	6.57 ± 2.25	Minimal change disease	1.74	NR	Fu: 2 mg/kg A: 0.5 g/kg 20% salt-poor soln	Reduction in body wt and abdominal circumference
Dharmarajy *et al.* [16]	Randomized cross-over trial	16	3–18	NS	1.3 g/dL	0.6 mg/dL	Fu: 1 mg/kg followed by 0.3 mg/kg for 24 h A: 1 g/kg 20% soln	Improvement in both diuresis and natriuresis

RCT—randomized control trial; NA—not available; NR—not reported; TDS—total dissolved solids; * denotes statistically significant findings (*p* < 0.05).

The effect of albumin and furosemide combination was also studied in children with nephrotic syndrome. An early report by Weiss *et al.* [13] demonstrated weight reduction with the combination of albumin and furosemide in 24 children with nephrotic syndrome.

In this study, infusion of either albumin or furosemide was compared with combination therapy. This was followed by a study of Haws and Baum [14] in 21 children. Each patient received an average of five infusions per hospitalization. Combination therapy caused an average of 1.2% reduction in body weight per infusion, suggesting a total of 6% weight reduction per hospitalization. Interestingly, the weight loss persisted in those children who were improving their proteinuria, but did not sustain their weight loss in those who were proteinuric. This study, therefore, suggests that the combination of albumin and furosemide therapy is effective only in patients whose nephrotic syndrome is in remission.

In another study by Bircan *et al.* [15], 14 children with nephrotic syndrome secondary to minimal change disease were studied. Combination therapy resulted in a decrease in body weight, abdominal ircumference, and edema 1 and 24 h after administration. However, this beneficial effect was transient.

In another study, Dharmaraj and colleagues [16] evaluated 16 children with nephrotic syndrome and refractory edema in a randomized cross-over trial to receive either combination therapy or furosemide alone. Urine output, urinary sodium, urinary chloride, urinary potassium, urinary osmolality, urinary osmolal and free water clearances were measured 3, 6, 12, and 24 h after infusions. The results from this study suggest a short-term positive effect of the combination therapy on diuresis and natriuresis.

Although all of the above studies suggest a transient beneficial effect of albumin and furosemide combination therapy, the report by Kapur and associates [17] did not find any effect of combination therapy compared to diuretic (combination of furosemide and spironolactone) therapy alone. Table 1 summarizes the findings of various studies in both adults and children.

## 5. Discussion

Various clinical trials regarding the use of albumin and furosemide to treat edema in patients with nephrotic syndrome have been published both in adults and children in an attempt to clarify if such a combination is beneficial in these patients [18,19]. Unfortunately a definitive recommendation regarding the use of albumin and furosemide has not been established given the variation in selection criteria, experimental design, and clinical endpoints.

An important consideration regarding selection criteria that deserves particular mention is defining diuretic resistance and establishing which patients meet criteria in order to draw appropriate and clinically relevant conclusions. This point was highlighted by Eadington *et al.* [7] in a Letter to the Editor that was published shortly after the Akcicek *et al.* [8] report. Akcicek *et al.* [8] findings were strongly challenged regarding patient selection. Eadington *et al.* [7] argued that a relatively high dose of loop diuretic would not have much effect on natriuresis via volume expansion with albumin, particularly if these patients were not established as diuretic resistant (Akcicek *et al.* [8] did not state if their subjects had failed therapy with maximal dosing of intravenous diuretics prior to beginning the study). The authors concluded that in nephrotic patients meeting the criteria of diuretic resistance, that is failure of response to maximum dosing of intravenous diuretic alone or diuretic combinations, the use of albumin is a potential treatment option. Interestingly, despite this astute observation, all subsequent randomized controlled studies (see Table 1) have not addressed this fundamental issue. A standardized definition of diuretic resistance and application in choosing patients who meet this criterion is necessary. Failure to address this issue could account for the persistent controversy regarding the use of albumin and furosemide in the nephrotic syndrome. Also, clinical evaluation of underfilling or overfilling cause of nephrotic syndrome is essential to an understanding of diuretic resistance.

Experimental design with respect to drug dosages and methods of administration is a second important consideration. Dosages of furosemide ranged from 40 mg to 220 mg with concomitant equimolar concentrations of salt-poor albumin in all studies. Both albumin and furosemide were administered as a combination, or albumin preceding furosemide administration. The dosage of furosemide used is important, as higher doses could make the addition of albumin less effective. Method of administration of albumin in relationship to furosemide is also important. Albumin has been shown to exert maximal effect of intravascular volume expansion within 30 to 60 min of administration. The timing of administration with albumin prior to furosemide could potentiate greater increases in diuresis in albumin and furosemide versus furosemide alone, as demonstrated by the work of Na *et al.* [10] and should be considered as a treatment modality in patients with documented diuretic resistance.

Finally, the use of concrete clinical endpoints is essential. The above studies were chosen for their inclusion of objective measures (urine output, sodium excretion) as opposed to more subjective values (resolution of edema). Additional endpoints used in some studies (ANP, MAP/blood pressure, GFR, ERPF) for extrapolation of albumin’s effect on the pharmacokinetics of furosemide were not significant. Based on these results the clinical outcomes that should be focused on in future studies should include diuretic and natriuretic parameters (urine output and sodium excretion, respectively) as these markers have had positive results.

## 6. Conclusions

In conclusion, the use of albumin and furosemide remains a controversial therapeutic option in the management of edema in patients with the nephrotic syndrome. Such controversy stems from variability in selection criteria, experimental design, and clinical endpoints. However, we suggest that the treatment be individualized, and that combination therapy should be considered in those patients with documented diuretic resistance. Large-scale randomized control trials in patients with documented diuretic resistance are needed to make evidence-based recommendations regarding the use of albumin and furosemide.
